# Idiopathic Granulomatous Mastitis: A Rare Confrontation

**DOI:** 10.7759/cureus.19420

**Published:** 2021-11-09

**Authors:** Muhammad Usman Hashmi, Ayousha Masood, Sobia Yaseen, Huzaifa Azam

**Affiliations:** 1 Department of Internal Medicine, Nishtar Medical University Hospital, Multan, PAK; 2 Department of Internal Medicine, Faisalabad Medical University Hospital, Faisalabad, PAK; 3 Department of Obstetrics and Gynaecology, Jinnah Hospital, Lahore, PAK; 4 Department of Anesthesiology, The Children Hospital and The Institute of Child Health, Multan, PAK

**Keywords:** breast pathology, breast abscess, suppurative breast lesion, tuberculous mastitis, breast cancer, mastitis, idiopathic granulomatous mastitis

## Abstract

Idiopathic granulomatous mastitis is a benign chronic inflammatory condition of the breast, the etiology of which has not been identified yet; it mimics two common breast disorders: breast carcinoma and tuberculous mastitis. Hence, this clinical entity poses difficulties in the diagnostic work-up. As clinical presentation and imaging findings often simulate other infectious and neoplastic etiologies, an accurate and early diagnosis is crucial to prevent misdiagnosis. Clear guidelines have yet to be established regarding treatment. In this report, we describe a case in which a patient presented with a painful breast mass and was diagnosed with idiopathic granulomatous mastitis after histological evaluation. We managed this patient with a combination of surgical excision and, subsequently, a course of antibiotics and steroids. To conclude, idiopathic granulomatous mastitis must be considered a possible differential while treating a patient with a lump in the breast tissue.

## Introduction

Idiopathic granulomatous mastitis is a rare but benign chronic inflammatory condition of the breast that often affects women of childbearing age. Several factors have been identified as risk factors for idiopathic granulomatous mastitis such as oral contraceptive pills (OCPs), autoimmunity, infectious agents, and spillage of milk from the breast lobules [1-3]. Moreover, pregnancy, breastfeeding, hyperprolactinemia, and Alpha-1 antitrypsin deficiency have been associated with the risk of the development of idiopathic granulomatous mastitis [3-4]. The disease mainly presents as a hard lump that grows over time. Other presentations can be local pain, inflammation, tumorous induration, skin ulceration, fistula formation, and nipple retraction. These presentations resemble cancer so early diagnosis and treatment are vitally important, especially to differentiate it from breast cancer of inflammatory variant [3-4]. There is no pathognomonic feature on USG, mammography, and MRI [1]. The disease is diagnosed on histological sections showing non-caseating granulomas, and in most of the cases, this is a diagnosis of exclusion [4]. As the name indicates, it is of an idiopathic etiology, therefore it poses a great dilemma in its treatment. Thus, there is no single well-established definitive cure or therapeutic strategy. However, a review of the literature reveals that surgical wide excision, corticosteroid therapy, colchicine, methotrexate, and azathioprine can be used for its treatment [1-2]. In this case report, we described a patient, suffering from idiopathic granulomatous mastitis for many weeks. Her disease remained undiagnosed for a considerably long period despite seeking healthcare advice. This resulted in prolonged physical, mental, and economic burdens on the patient because delayed diagnosis hampered the initiation of optimal therapy. Hence, the purpose of reporting this case is to educate and raise awareness among practicing physicians regarding this rare clinical entity. Thus, we can avoid unnecessary mastectomies, which are still being reported [5].

## Case presentation

**Figure 1:**
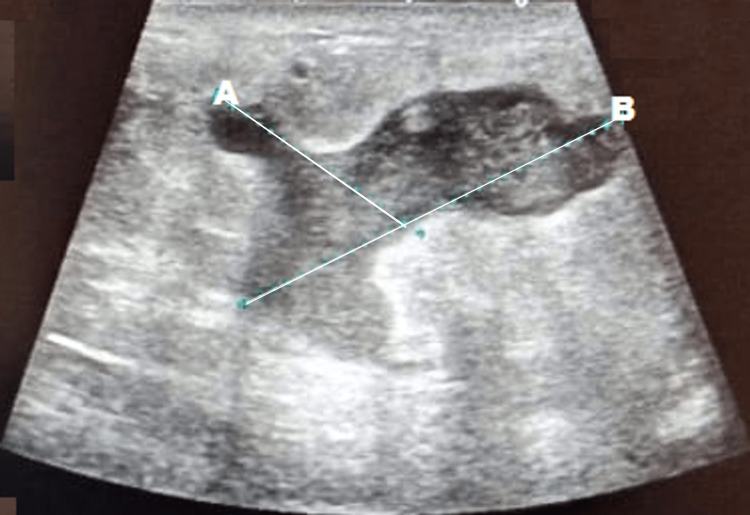
Ultrasonography of the affected breast which demonstrated a lesion in the parenchyma with thin fluid streaks. These findings are marked by the white lines A and B.

A 29-year-old, non-lactating, and non-gravid woman presented with a complaint of a lump in her right breast. The patient also complained of low-grade fever and unilateral pain in breast tissue. The general physical examination showed a one-centimeter erythematous and tender mass in the right breast tissue. There was no nipple discharge, axillary lymphadenopathy, or external draining sinuses. The primary care physician evaluated the patient and called for a USG for the assessment of the affected breast, which demonstrated an ill-defined lesion with thin fluid streaks in the lower outer quadrant, suggesting an inflammatory lesion (Figure 1).

**Figure 2:**
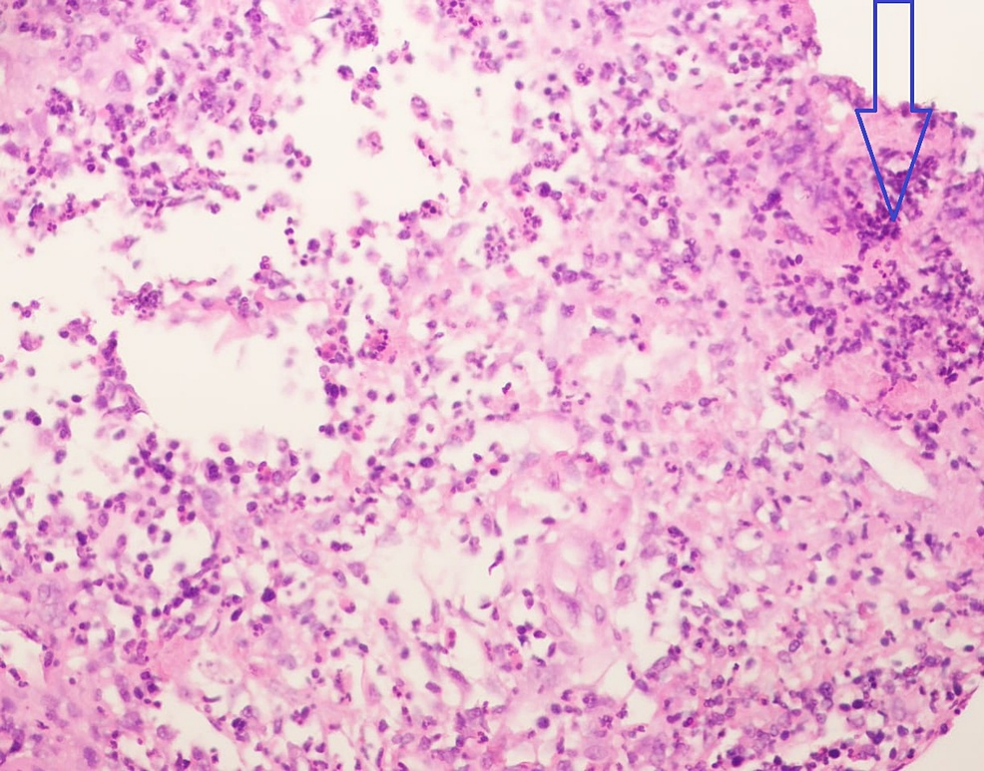
Histopathological findings of acute mastitis demonstrating areas of acute inflammation. Polymorphonuclear cells seen in acute inflammation are depicted by an unfilled blue arrow.

At the same time, cystic fluid from her breast was aspirated, and the bacterial culture test showed no growth of any microorganisms after 48 hours. The cytology revealed predominantly neutrophils and degenerating cells in a hemorrhagic background, which suggested an acute suppurative inflammatory process of the affected breast as shown in Figure 2.

**Figure 3:**
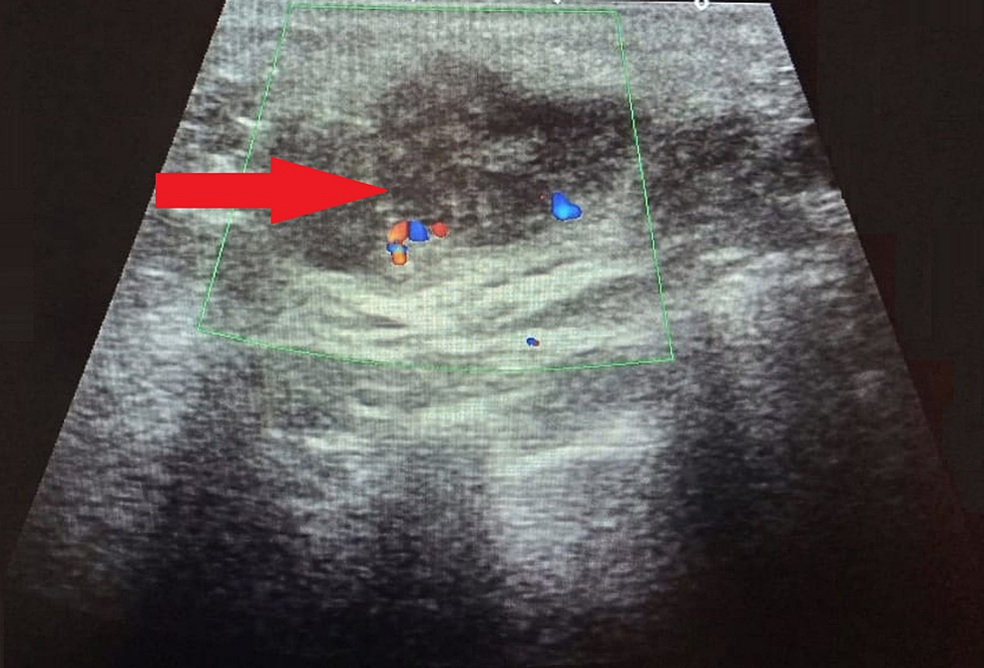
Ultrasonography of the breast showing an ill-demarcated hypoechoic irregular lesion with no fluid components. The area of pathology is marked by a solid red arrow.

Based on the clinical features, imaging findings, and cytology reports, a presumptive diagnosis of acute mastitis with underlying bacterial abscess was established. Subsequently, she was being prescribed a course of antibiotics and antipyretics for one week. However, instead of improving, her condition worsened over time. Therefore, the physician decided to repeat the ultrasound-guided fine-needle aspiration and cytology (FNAC). The sonomammogram of the right breast revealed an ill-demarcated hypoechoic irregular lesion involving the parenchyma of the right breast in the outer lower and adjoining upper quadrants. The lesion measured 34.4 millimeters on a long axis with no fluid components as shown in Figure 3.

**Figure 4:**
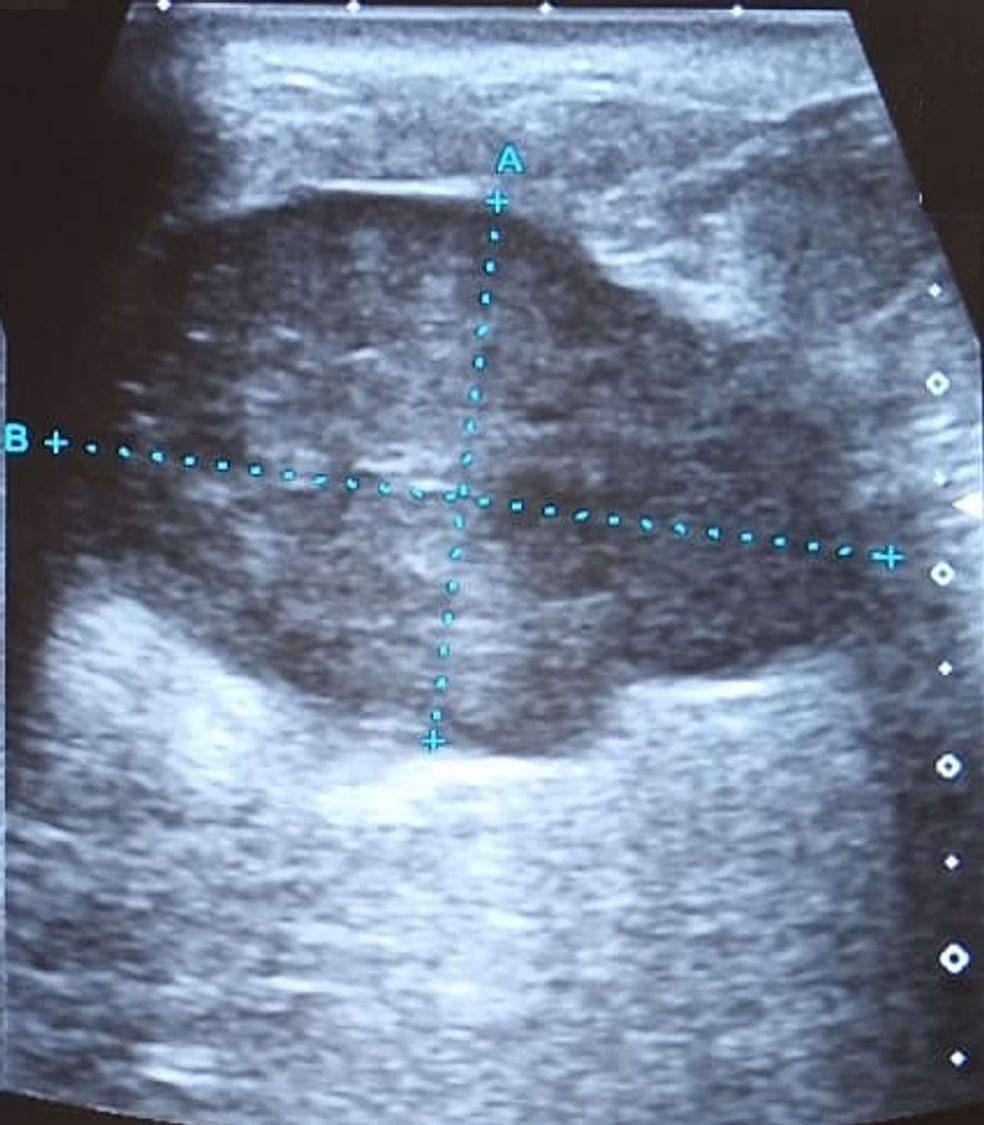
Ultrasonography of the breast indicated an ill-demarcated hypoechoic irregular lesion with thin fluid streaks as marked by the dotted lines A and B.

The repeated cytological examination also showed cellular clusters comprising histiocytes and epithelioid-like cells. Hence, based on these findings, the treating physician concluded that the ongoing clinical picture was more suggestive of tuberculosis of the breast. Therefore, after discussing with the patient, a therapeutic trial of an anti-tuberculosis therapy (ATT) was prescribed for four weeks. However, instead of any clinical improvement, her condition worsened over time and she had to visit her physician again just after three weeks. Her re-assessment by sonomammography was performed, which indicated an ill-demarcated hypoechoic irregular lesion, measuring 44.7×35.4 millimeters in the outer lower and adjoining upper quadrants of the right breast with thin fluid streaks as shown in Figure 4.

**Figure 5:**
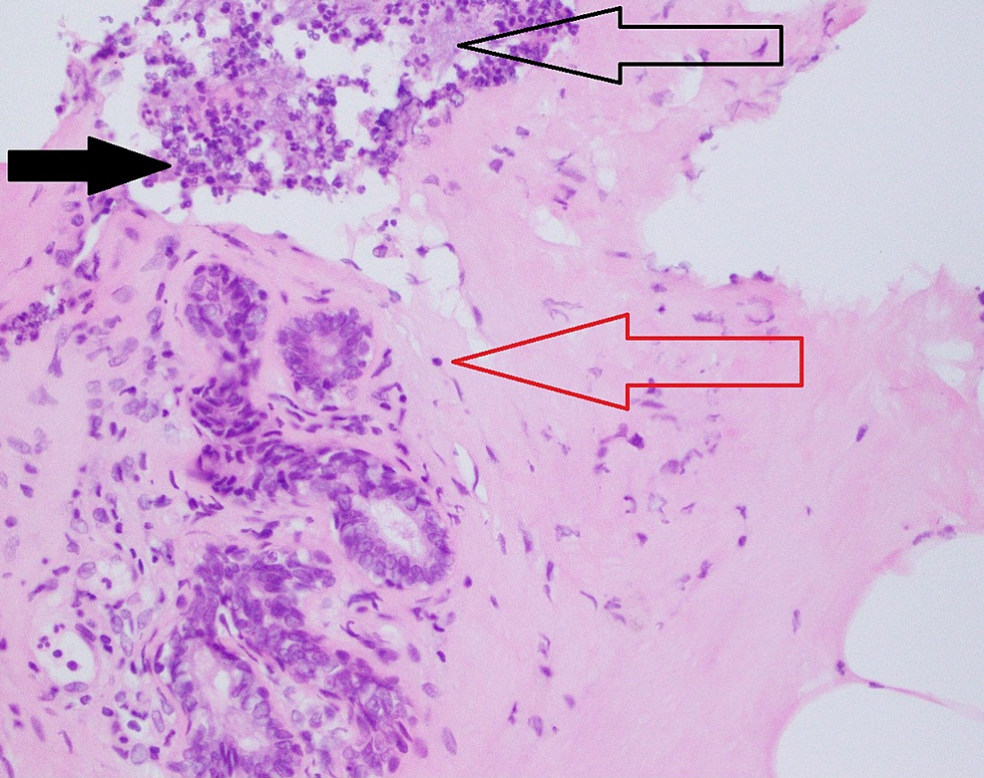
Histopathology of breast parenchyma exhibiting dense areas of acute and chronic inflammation along with predominant lobulocentric granulomatous inflammation. The solid black arrow shows neutrophils with abscess formation, whereas the unfilled black arrow marks the histiocytes. The lymphocytes around the ducts are marked by the red unfilled arrow.

The sonologist concluded that these findings were consistent with an infectious or inflammatory lesion. Afterward, the primary care physician referred her to a teaching hospital for a surgical consult. So, re-evaluation of the patient was performed by a detailed history, thorough general physical examination, and routine laboratory investigations. It was then planned to perform incision and drainage of pus collection and excisional tissue biopsy after surgical resection of the mass. The excisional biopsy material was sent for histopathology and pussy discharge for acid-fast bacilli (AFB) smear. After surgery, she received broad-spectrum antibiotics (ceftriaxone and moxifloxacin) for two weeks. The subsequent histopathology showed areas of acute and chronic inflammation along with predominant lobulocentric granulomatous inflammation as shown in Figure 5.

These findings were suggestive of idiopathic granulomatous mastitis as an etiology. Concurrently, the AFB smear also came out to be negative. Hence, a final diagnosis of idiopathic granulomatous mastitis was established. Afterward, corticosteroids (prednisolone) were also added to the drug dosage regimen of this patient. The patient was followed up after one month, and during this period her symptoms had started to resolve. The medical team agreed to advise her to follow up until the complete resolution of her disease, and she was followed up monthly for the next three months. During these follow-up visits, we evaluated the patient for improvement in the clinical symptoms. Finally, a USG scan was performed, which showed complete resolution of the lesion with no abnormal findings. Table 1 describes the timeline of all these events.

**Table 1:** Timeline for the case report showing the chronology of the various events. CBC: Complete blood count; ESR: Erythrocyte sedimentation rate; FNAC: Fine-needle aspiration and cytology; AFB: Acid-fast bacilli

Dates	Summaries of initial and follow-up visits	Diagnostic testing	Interventions
April 25, 2020	Initial visit at a primary healthcare physician clinic with the above-mentioned complaints. On examination, there was a one cm right breast mass with surrounding erythema. There was no nipple discharge, axillary lymphadenopathy, or external draining sinuses. The initial diagnosis was acute mastitis with underlying bacterial abscess.	CBC showed mild leukocytosis. ESR was raised. USG revealed an inflammatory breast lesion. FNAC showed an acute suppurative inflammatory process.	Oral antibiotics, antipyretics.
May 2, 2020	No clinical improvement, rather symptoms worsened over time	CBC showed normal leukocyte counts. ESR was elevated. USG guided FNAC showed cellular clusters comprising of histiocytes and epithelioid-like cells.	Anti-tuberculosis therapy was prescribed for four weeks.
May 21, 2020	Fever and lump did not improve, rather they worsened and prompted another visit.	Routine laboratory investigations were normal except ESR, which was elevated. USG demonstrated an infective/inflammatory lesion (measuring 44.7×35.4 mm with thin fluid streaks).	The patient was referred to the tertiary care hospital for a surgical consult.
May 25, 2020	The patient was re-evaluated and surgery was planned for the next day. She had an uneventful recovery and was discharged on the third postoperative day.	Histopathology of excisional biopsy and acid-fast bacilli smear.	Incision and drainage of the pus collection and excisional tissue biopsy after surgical resection of the mass. Broad-spectrum antibiotics (ceftriaxone and moxifloxacin) for two weeks.
June 3, 2020	The first follow-up visit after surgery: AFB smear came out to be negative, histopathology revealed idiopathic granulomatous mastitis as likely etiology. Finally, a diagnosis of idiopathic granulomatous mastitis was established.		High-dose corticosteroids (prednisolone) were started.
July 5, 2020	The second follow-up visit after surgery: symptoms started to improve.		High-dose corticosteroids
August 10, 2020	The third follow-up visit after surgery: symptoms continued to resolve gradually.		High-dose corticosteroids
September 12, 2020	The fourth follow-up visit after surgery: complete resolution of symptoms with no recurrence.	USG scan showed complete resolution of the lesion with no abnormal findings.	Corticosteroid therapy was discontinued.

## Discussion

Idiopathic granulomatous mastitis is an uncommon pathological process involving breast tissue. Owing to the variety and non-specificity in the spectrum of clinical manifestations, this disease has been quite a diagnostic challenge for healthcare professionals. Therefore, many a time this debilitating condition remains undiagnosed or misdiagnosed, causing unnecessary investigations and procedures. This puts patients at risk of functional impairment along with an economic burden. The current case was initially diagnosed as acute mastitis with an underlying breast abscess and afterward, it was falsely labeled as tuberculosis of the breast. It is noteworthy that these conditions are among common differential diagnoses of idiopathic granulomatous mastitis. During the early course of the disease, the patient received antibiotics and antituberculosis therapy (ATT), which is similar to the findings of a detailed study by Mahmodlou R et al. in which two out of 46 patients had ATT as part of the management [2]. It is worthy to note that this patient belonged to the tuberculosis (TB) endemic region, so an anchoring bias might have played a role in the misdiagnosis of breast TB. In addition to it, Seo HR et al. have studied the differentiating features between TB of breast and idiopathic granulomatous mastitis [6]. This study found statistically significant clinical differences between the breast TB and idiopathic granulomatous mastitis patient groups. These differentiating features include older age in breast TB as compared to idiopathic granulomatous mastitis (40 vs 33.5 years, p =.018), more frequent axillary lymphadenopathy in the tuberculous mastitis group (50% vs 20.6%, p =.048), and a history of tuberculosis of lungs in patients with tuberculosis of breast [6]. Interestingly, our case also presented in the younger age group and did not have axillary lymphadenopathy. Moreover, the absence of lymphocytosis on complete blood count (CBC) was against the diagnosis of tuberculosis.

Establishing the diagnosis of idiopathic granulomatous mastitis is a complex and laborious task that involves a series of investigations. The ultrasonography of the breast proved its significance and accuracy in the workup of this complicated and rare clinical entity. A study by Hasbahceci M et al. revealed that a hypo-echoic or heterogeneous mass with or without tubular extensions is the most frequent finding on sonomammography of the breast. This study concluded that, in the case of an inflammatory lesion in the breast, heterogeneity of the breast parenchyma and abscess formation was likely to be the cardinal features of idiopathic granulomatous mastitis [7]. These radiological features are also consistent with the sonomammography findings of our patient.

Regarding treatment, our patient showed a dramatic response to high-dose corticosteroids (prednisolone). This supports the findings of a previous research study, emphasizing a higher success rate with lower recurrences by using high-dose steroids [8]. It is also proposed that steroids possibly reduce the need for surgery [9]. Hence, Montazer M et al. reported that the patients who received a high dose of corticosteroids had a significantly higher rate of remission compared to the patients who received a low dose of corticosteroids (93.3% vs. 53.3%, p=0.03) [8]. Interestingly, among patients with remission, recurrence was also significantly lower in the high dose compared to low dose prednisolone (0% vs. 37.5%, p=0.04) [8].

Therefore, systemic corticosteroids and antibiotics are the most frequently used agents in the medical treatment of idiopathic granulomatous mastitis. However, immunosuppressant agents such as methotrexate and azathioprine can also be used for the management of idiopathic granulomatous mastitis. Furthermore, we can opt for a therapeutic trial of bromocriptine and anti-inflammatory agents (colchicine) in combination with steroids [10-11]. In cases, where medical management fails, surgical intervention should be the next best step. This includes abscess drainage, local and wide excisions, or even mastectomy when there is no significant remission after a long time of conservative management [12].

## Conclusions

Idiopathic granulomatous mastitis is an exceedingly rare pathology of breast tissue. Owing to non-specific and ambiguous clinical features, it can remain undiagnosed or misdiagnosed, which leads to an undue delay in the initiation of treatment. The sonomammography of the breast is the cornerstone of its diagnostic work-up. In case of an inflammatory lesion of the breast, a hypoechoic or heterogeneous mass with or without tubular extensions on sonomammography should raise the suspicion of idiopathic granulomatous mastitis.
